# Gastrointestinal Stromal Tumours (GISTs) with KRAS Mutation: A Rare but Important Subset of GISTs

**DOI:** 10.1155/2023/4248128

**Published:** 2023-08-24

**Authors:** Dorinda Mullen, Rajkumar Vajpeyi, Jose-Mario Capo-Chichi, Klaudia Nowak, Newton Wong, Runjan Chetty

**Affiliations:** ^1^Division of Anatomical Pathology, Laboratory Medicine Program, University Health Network, University of Toronto, Toronto, Canada; ^2^Department of Clinical Laboratory Genetics, Laboratory Medicine Program, University Health Network, University of Toronto, Toronto, ON, Canada; ^3^Department of Cellular Pathology, Southmead Hospital, Bristol, UK; ^4^Deciphex, Dublin, Ireland

## Abstract

Gastrointestinal stromal tumours (GISTs) are the most common mesenchymal tumours of the GI tract, usually found in the stomach, jejunum, and ileum. Typically, they are *KIT* or *PDGFR*-mutated, allowing for targetable treatments with tyrosine kinase inhibitors such as imatinib. Here, we present two *KRAS*-mutated wild-type gastrointestinal tumours (GISTs). Both cases occurred in the small bowel of females. Immunohistochemical studies on both tumours showed KIT and DOG-1 positivity, with SDHB retained. Molecular analysis revealed a *KRAS G12D* mutation and a *KRAS G13D* mutation, respectively. Wild-type GISTs are extremely uncommon. They typically occur in the stomach or the small bowel. *KRAS* is one of the genes implicated in this subset of GIST, with *KRAS G12D* being the most frequently encountered mutation. GIST *KRAS* mutations can arise alone or in conjunction with *KIT, PDFRA*, or *BRAF* mutations. Identification of these rare molecular subtypes is clinically important due to their resistance to imatinib therapy.

## 1. Introduction

Gastrointestinal stromal tumours (GISTs) are an established tumour group with the vast majority possessing characteristic morphology, immunohistochemical profiles, molecular findings, and specific targeted therapeutic agents. A small subset of GISTs fall outside of the usual molecular paradigm of *KIT* or *platelet-derived growth factor alpha* (*PDGFRA*) or *succinate dehydrogenase (SDH)* mutations which account for 85% of cases. Tumours lacking theses mutations are regarded as wild-type GIST. Of the remaining 15% of GISTs, most will harbour *neurofibromatosis-1* (*NF-1*) or *second RAF paralogue* (*BRAF*) mutations. The least frequently encountered are those bearing a *Kirsten rat sarcoma (KRAS)* mutation. Although this molecular scenario is extremely uncommon, its awareness is important because of the therapeutic ramifications for patients.

We present two sporadic GIST cases highlighting the practical issues associated with wild-type GIST, review the pertinent literature on this unusual GIST molecular scenario, and provide a comprehensive summary of the immunohistochemical-molecular associations in GIST.

## 2. Materials and Methods

Specimens were received in 10% neutral buffered formalin, and 10 sections were taken from the tumour. These were processed in a routine manner generating haematoxylin and eosin-stained sections.

Immunohistochemistry was performed on the formalin fixed, paraffin embedded tissue for KIT (CD117), DOG-1, CD34, desmin, caldesmon, smooth muscle actin, S100, cytokeratin AE1/3, and succinate dehydrogenase (SDHB). Appropriate positive and negative controls were run in parallel.

### 2.1. Molecular Analysis for Case 1

Mutational analysis by next generation sequencing (NGS) was performed using the TruSight-15 panel (Illumina) that targets entire coding regions or hotspot sequences of 15 genes including the following: *KIT, PDGFRA, BRAF, NRAS, KRAS, PIK3CA, AKT1, EGFR, ErbB2, RET*, and *p53*. The NGS panel was run on two different sampled areas of the tumour.

Specific to *KIT* and *PDGFRA*, the NGS panel covered the following exons:

*KIT*: exons 8–*11*, 13-14, and 17-18 and *PDGFRA*: exons 12, 14, and 18.

### 2.2. Molecular Analysis for Case 2

Sanger sequencing was performed on *KIT* exons 9, 11, 13, and 17 and *PDGFRA* exons 12, 14, and 18; pyrosequencing was performed on *BRAF* exons 11 and 15; real time amplification refractory mutation system polymerase chain reaction (ARMS PCR) was performed for *PIK3CA* exons 9 and 20 and pyrosequencing was performed covering *KRAS* and *NRAS* codons 12, 13, 59, 61, 117, and 146.

## 3. Case Summary

### 3.1. Case 1

A 77-year-old female with a history of squamous cell carcinoma of the perianal region that was treated with chemoradiation was noted to have a 6 cm solid-cystic mass in the right pelvic region on surveillance MRI. She did not have any other relevant clinical history, and there was no family history of malignancies or neurofibromatosis type 1.

**Figure 1:**
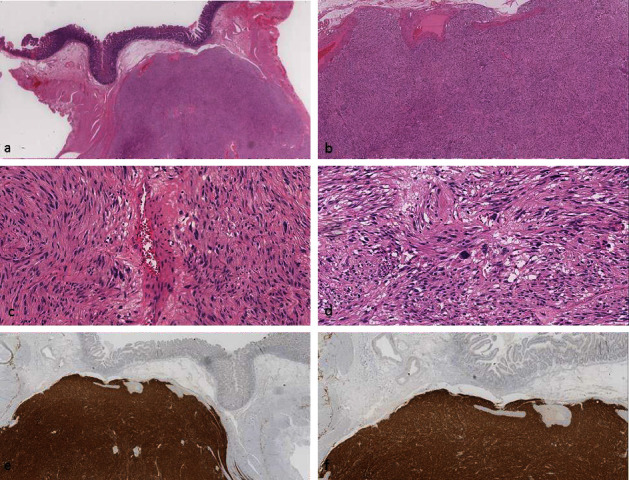
Case 1: the tumour formed a well-circumscribed, intramural mass with intact overlying mucosa (a). It was composed of spindle and epithelioid cells arranged in interlacing fascicles (b). Occasional bizarre, larger multinucleated cells were also present scattered throughout the lesion (c). Focally, there were areas of oedema and microcystic change (d). Immunohistochemistry showed intense, diffuse positivity for CD117 (KIT) (e) and DOG-1 (f).

The mass arose from the ileum and was densely adherent to the uterus. Histological sections demonstrated a well-circumscribed neoplasm arising from the muscularis propria composed of spindle cells with eosinophilic cytoplasm arranged in fascicles. There was moderate nuclear pleomorphism with scattered multinucleated and giant cells. Oedema, microcystic change, and cystic degeneration were also noted (Figure 1). The mitotic rate was 2 per 5 mm^2^. There was no necrosis, infarction, or lymphovascular invasion. Immunohistochemistry showed strong and diffuse positivity for CD117, DOG-1, and CD34 and negative for S100, SMA, desmin, caldesmon, and pankeratin. There was retention of staining with SDHB immunohistochemistry. Molecular studies identified a *KRAS p.Gly12Asp* (commonly known as *G12D*) activating mutation at an allelic frequency of 79%.

A final diagnosis was of a *KRAS G12D*-mutated, moderate risk GIST of small bowel origin. There was no role for adjuvant imatinib once the molecular profile of the GIST was known. The treatment plan was for ongoing surveillance with imaging of the abdomen and pelvis every 3 months. Follow-up scans have been negative to date. The patient continues to be well.

### 3.2. Case 2

A 50-year-old female presented with left iliac fossa pain and weight loss. She was found to have iron deficiency anaemia. Imaging revealed loops of the small bowel around a soft tissue lesion. Grossly, the tumour measured 12 cm and had a grey cut surface with focal cystic change and haemorrhage. Morphologically, it consisted of a mixture of spindle and epithelioid cells. The mitotic rate was 30 per 5 mm^2^. Immunohistochemistry revealed diffuse positivity for CD117, DOG-1, and CD34, focal SMA positivity, and negative for S100 and desmin. The tumour retained SDHB expression. Molecular studies identified a *KRAS p.Gly13Asp* (commonly known as *G13D*) activating mutation.

A final diagnosis was of a *KRAS G13D*-mutated, high risk, mixed-cell type GIST of small bowel origin. Unfortunately, no follow-up information is available on this patient.

Thus, both these GISTs were wild-type (intact *KIT*, *PDGFRA*, and SDH genes or SDH-competent) GISTs with a *KRAS* mutation.

## 4. Discussion

85–90% of GISTs will harbour a mutation in either one of the receptor tyrosine kinase genes *KIT* or *PDGFRA*. In routine practice, most mutational analyses are geared towards seeking activating mutations in these two genes. In approximately 15% of GISTs, both *KIT* and *PDGFRA* genes are found to be intact and so is referred to as a *KIT/PDGFRA*wild-type GIST. This molecular profile is most frequently encountered in paediatric GISTs, and when seen in adult patients, it is sometimes referred to as paediatric-like GIST [1–4]. In wild-type GISTs, the vast majority (80%) will have a mutation or epimutation in the *SDH* complex of genes. These GISTs are characterised by SDH protein deficiency and are also referred to as SDH-deficient GISTs. In the remaining 20% of wild-type GISTs, the *SDH* gene complex is also normal or wild type, resulting in the so called *KIT/PDGFRA/SDH* wild type or SDH-competent GIST. The next most frequent genes involved in *KIT/PDGFRA/SDH*wild-type GIST are *NF-1* and *BRAF* [5, 6]. Although *NF-1* mutations were not sought in either of the cases presented herein, there was no family history or clinical stigmata to suggest that they were neurofibromatosis patients.

There are approximately 1% of GISTs that are wild type for *KIT, PDGFRA, SDH, NF-1*, and *BRAF*. A plethora of genes have been implicated in this particular subset of GISTs such as *PIK3CA, NRAS, HRAS*, and *KRAS.* Rare examples described in the literature include alterations and point mutations in the fibroblast growth factor/receptor (FGF/FGFR) signalling pathway and gene fusions such as *ETV6-NTRK*, *FGFR1-TACC1*, and *CDC42BPB- ALK* [7–11].

Following next generation sequencing analysis, our two cases fall into this last category of GIST and are characterised by *KRAS* mutations. *KRAS*-mutated GISTs share morphological and immunohistochemical properties with *KIT/PDGFRA*-mutated GISTs. In addition to the spindle and/or epithelioid cell morphology, these GISTs are also KIT and DOG-1 positive by immunohistochemistry (Table 1). Thus, if mutational analysis is restricted only to activating mutations in *KIT, PDGFRA, SDHB*, and *BRAF*, this rare subgroup of GISTs would not be detected. Furthermore, it is also known that *KRAS*-mutated GISTs are resistant to first line receptor tyrosine kinase inhibitors and so are clinically relevant.

As a group, *KRAS*-mutated GISTs are exceedingly rare and thought to account for 0.2% of all GISTs, although a detailed molecular analysis of 514 cases failed to detect any cases with *KRAS* mutations [12].

Table 2 highlights the pertinent literature and findings of *KRAS*-mutated GISTs. In 2012, Miranda et al. [13] highlighted 3 cases of GIST with *KRAS* and coexistent *KIT* (2 cases) and *PDGFRA* (1 case). One of the cases contained two separate *KRAS* mutations. Two cases were in the stomach, and a case with *KRAS* and *PDGFRA* mutations was in the small bowel. The *KRAS* mutations occurred at the following residue positions: G12D, G13D, G12A, and G12D. No details on demographics, histological features, and immunohistochemistry were provided.

Antonescu et al. [14] also encountered a GIST with concomitant *KRAS* and *KIT* mutations in the small bowel of a 66 -year-old male. The tumour was described as anaplastic with rhabdoid cells and was negative for KIT by immunohistochemistry although the pretreated GIST was not stained. The patient also received preoperative imatinib. The *KRAS* mutation occurred at residue G12V.

Serrano et al. [15] also encountered a *KRAS*- and *KIT*-mutated GIST in the stomach of a 61-year-old male. The GIST had spindle cell morphology and was *KIT* positive by immunohistochemistry, and the *KRAS* mutation was at residue G12R.

The case described by Hechtman et al. [16] was a *KRAS*-only mutated GIST. It arose in the stomach of a 67-year-old man, was positive for KIT immunohistochemistry, and had lost SDHB protein expression. There is limited information on the one case described by Gao et al. [17] with a *KRAS* only mutation except that the mutation occurred at residue G13D. They also encountered other KRAS mutations in combination with BRAF mutations [17].

A *KRAS*-only mutated GIST was described by Mavroeidas et al. [18] describes in a male patient with a G12D KRAS mutation in his gastric GIST. The patient had a subtotal gastrectomy, was treated with imatinib but relapsed quickly, and died within 15 months of diagnosis.

Thus, *KRAS* mutations in GIST occur in two distinct scenarios as follows:

- *KRAS* mutations coexisting with other mutations such as *KIT, PDGFRA,* and *BRAF.* This appears to be the commoner scenario in which *KRAS* mutations occur in GISTs.
- *KRAS* mutations only/exclusively.

The *KRAS* gene is one of the most frequently mutated genes in human cancers. Within the gene, activating mutations are most often seen at residue positions 12, 13, and 61, with the G12 residue being mutated 80% of the time [19]. Of these, G12 mutates to aspartate (G12D) in 36% of cases, to valine (G12V) in 23%, and cysteine (G12C) in 14% [19].

*KRAS* is located downstream to both *KIT* and *PDGFRA,* and there is no obvious explanation as to why both KIT and DOG1 immunohistochemistry are positive in a GIST harbouring a *KRAS* mutation. Since only specific exons in the *KIT* and *PDGFRA* genes were examined by NGS, it remains possible that other untested exons of the genes may have been mutated resulting in protein overexpression. In the absence of this, a possible feedback loop between the RAS signal transduction pathway and *KIT* may result in KIT protein overexpression.

## 5. Conclusion

This overview highlights a rare cohort of wild-type GISTs that bear *KRAS* mutations. While being distinctly rare, they are important to recognise from a therapeutic and prognostic point of view as the *KRAS* mutation confers resistance to imatinib therapy. Furthermore, our case report raises the issue as to whether all GISTs should have reflex molecular testing rather than relying on immunohistochemistry only.

In these two particular cases, based on the immunohistochemical results (Table 1) solely, it may have been assumed that the GISTs contain a *KIT* or *PDGFRA* mutations. If this did indeed occur, the patient would have been treated, unsuccessfully, with imatinib. Performing a full molecular analysis to detect a rare (1% of cases) subset of GISTs has to be balanced with the cost associated with this approach.

## Figures and Tables

**Table 1:** Immunohistochemical molecular scenarios in gastrointestinal stromal tumours (GISTs).

Scenarios	% of GIST	Immunohistochemistry			Molecular					
		KIT	DOG1	SDHB	KIT	PDGFR*α*	SDH	BRAF	NF1	Other
(i) Usual GIST	85–90%	Pos	Pos	Retained	Mutated^*∗*^	Mutated^*∗*^	Wt	Wt	Wt	Wt
(ii) KIT/PDGFR*α*SDH-deficient GIST	10–15%	Pos	Pos	Lost	Wt	Wt	Mutated or methylated	Wt	Wt	Wt
(iii) KIT/PDGFR*α*/SDH GIST	1-2%	Pos	Pos	Retained	Wt	Wt	Wt	Mutated^*∗∗*^	Mutated^*∗∗*^	Wt
(iv) KIT/PDGFR*α*/SDH GIST	<1%	Pos	Pos	Retained	Wt	Wt	Wt	Wt	Wt	KRAS/PIK3C/NTRK/FGFR etc. mutated
(v)	<1%	Neg	Neg	Retained	Mutated^*∗*^	Mutated^*∗*^	Wt	Wt	Wt	Wt
(vi)	Rare	Neg	Pos	Retained	Wt	Mutated^*∗*^	Wt	Wt	Wt	Wt
(vii) ^*∗∗∗*^	Very rare	Neg	Neg	Retained	Wt	Wt	Wt	Wt	Wt	Wt

Pos = positive; Neg = negative; Wt = wild type; ^*∗*^ = KIT or PDGFR*α* is mutated;  ^*∗∗*^ = BRAF or NF1 is mutated; ^*∗∗∗*^ = this is a very rare scenario, and all tests should be repeated. Consideration whether this is in fact a GIST should be entertained.

**Table 2:** *KRAS*-mutated GIST.

References	Age/gender	Site	*KRAS* mutation	KIT IHC	DOG1 IHC	SDH IHC	Histology
Miranda et al. [13]	NM	Gastric	G12D^*∗*^	NM	NM	NM	NM
Miranda et al. [13]	NM	Small bowel	G13D^*∗∗*^	NM	NM	NM	NM
Miranda et al. [13]	NM	Gastric	G12A/G13D^*∗*^	NM	NM	NM	NM
Antonescu et al. [14]	66/male	Small bowel	G12V^*∗*^	Neg	NM	NM	Anaplastic
Serrano et al. [15]	61/male	Gastric	G12R^*∗*^	Pos	NM	NM	Spindle
Hechtman et al. [16]	67/male	Gastric	G12V	Pos	NM	Lost	Pleomorphic epithelioid
Gao et al. [17]	NM	NM	G13D	NM	NM	NM	NM
Mavroeidas et al. [18]	NM/male	Gastric	G12D	NM	NM	NM	Spindle
Current case 1	77/female	Small bowel	G12D	Pos	Pos	Retained	Pleomorphic spindle
Current case 2	50/female	Small bowel	G13D	Pos	Pos	Retained	Epithelioid and spindle

IHC = immunohistochemistry; Pos = positive; Neg = negative; NM = not mentioned; ^*∗*^ = with concomitant KIT mutation; ^*∗∗*^ = with concomitant PDGFRA mutation.

## Data Availability

The data used to support the findings of this study are available from the corresponding author upon request.

## References

[1] Szucs Z., Thway K., Fisher C. (2017). Molecular subtypes of gastrointestinal stromal tumors and their prognostic and therapeutic implications. *Future Oncology*.

[2] von Mehren M., Joensuu H. (2018). Gastrointestinal stromal tumors. *Journal of Clinical Oncology*.

[3] Boikos S. A., Pappo A. S., Killian J. K. (2016). Molecular subtypes of KIT/PDGFRA wild-type gastrointestinal stromal tumours: a report from the national institutes of health gastrointestinal stromal tumour clinic. *JAMA Oncology*.

[4] Italiano A. (2018). KIT and PDGFRA wild-type gastrointestinal stromal tumours (GISTs): ESMO biomarker factsheet. https://oncologypro.esmo.org/education-library/factsheets-on-biomarkers/kit-and-pdgfra-wild-type-gastrointestinal-stromal-tumours-gists.

[5] Hostein I., Faur N., Primois C. (2010). BRAFMutation status in gastrointestinal stromal tumors. *American Journal of Clinical Pathology*.

[6] Miettinen M., Fetsch J. F., Sobin L. H., Lasota J. (2006). Gastrointestinal stromal tumours in patients with neurofibromatosis 1: a clinicopathologic and molecular genetic study of 45 cases. *The American Journal of Surgical Pathology*.

[7] Mei L., Smith S. C., Faber A. C. (2018). Gastrointestinal stromal tumors: the GIST of precision medicine. *Trends in Cancer*.

[8] Brenca M., Rossi S., Polano M. (2016). Transcriptome sequencing identifies ETV6-NTRK3 as a gene fusion involved in GIST. *The Journal of Pathology*.

[9] Shi E., Chmielecki J., Tang C. M. (2016). FGFR1 and NTRK3 actionable alterations in Wild Type gastrointestinal stromal tumours. *Journal of Translational Medicine*.

[10] Huang W., Yuan W., Ren L. (2022). A novel fusion between CDC42BPB and ALK in a patient with quadruple wild-type gastrointestinal stromal tumor. *Mol Genet Genomic Med*.

[11] Astolfi A., Pantaleo M. A., Indio V., Urbini M., Nannini M. (2020). The emerging role of the FGF/FGFR pathway in gastrointestinal stromal tumour. *International Journal of Molecular Sciences*.

[12] Lasota J., Xi L., Coates T. (2013). No KRAS mutations found in gastrointestinal stromal tumors (GISTs): molecular genetic study of 514 cases. *Modern Pathology*.

[13] Miranda C., Nucifora M., Molinari F. (2012). KRAS and BRAF mutations predict primary resistance to imatinib in gastrointestinal stromal tumors. *Clinical Cancer Research*.

[14] Antonescu C. R., Romeo S., Zhang L. (2013). Dedifferentiation in gastrointestinal stromal tumour to an anaplastic KIT-negative phenotype: a diagnostic pitfall: morphologic and molecular characterization of 8 cases occurring either de novo or after imatinib therapy. *The American Journal of Surgical Pathology*.

[15] Serrano C., Wang Y., Mariño-Enríquez A. (2015). KRAS and KIT gatekeeper mutations confer polyclonal primary imatinib resistance in GI stromal tumors: relevance of concomitant phosphatidylinositol 3-kinase/AKT dysregulation. *Journal of Clinical Oncology*.

[16] Hechtman J. F., Zehir A., Mitchell T. (2015). Novel oncogene and tumor suppressor mutations inKITandPDGFRAwild type gastrointestinal stromal tumors revealed by next generation sequencing: novel Mutations in SDH Deficient Gists. *Genes Chromosomes & Cancer*.

[17] Gao J., Li J., Li Y. (2016). Intratumoral KIT mutational heterogeneity and recurrent KIT/PDGFRA mutations in KIT/PDGFRA wild-type gastrointestinal stromal tumors. *Oncotarget*.

[18] Mavroeidis L., Metaxa-Mariatou V., Papoudou-Bai A. (2018). Comprehensive molecular screening by next generation sequencing reveals a distinctive mutational profile of KIT/PDGFRA genes and novel genomic alterations: results from a 20-year cohort of patients with GIST from north-western Greece. *ESMO Open*.

[19] Vatansever S., Erman B., Gümüş Z. H. (2019). Oncogenic G12D mutation alters local conformations and dynamics of K-Ras. *Scientific Reports*.

